# Stem cell mobilization in multiple myeloma: challenges, strategies, and current developments

**DOI:** 10.1007/s00277-023-05170-0

**Published:** 2023-03-22

**Authors:** Xiaolei Wei, Yongqiang Wei

**Affiliations:** Department of Hematology, Nanfang Hospital, Southern Medical University, No. 1838 Guangzhou Avenue North, Guangzhou, 510515 China

**Keywords:** Multiple myeloma, Stem cell mobilization, Graft characteristics, Mobilization regimen

## Abstract

Among hematological malignancies, multiple myeloma (MM) represents the leading indication of autologous hematopoietic stem cell transplantation (auto-HCT). Auto-HCT is predominantly performed with peripheral blood stem cells (PBSCs), and the mobilization and collection of PBSCs are essential steps for auto-HCT. Despite the improved success of conventional methods with the incorporation of novel agents for PBSC mobilization in MM, mobilization failure is still a concern. The current review comprehensively summarizes various mobilization strategies for mobilizing PBSCs in MM patients and the evolution of these strategies over time. Moreover, existing evidence substantiates that the mobilization regimen used may be an important determinant of graft content. However, limited data are available on the effects of graft characteristics in patient outcomes other than hematopoietic engraftment. In this review, we discussed the effect of graft characteristics on clinical outcomes, mobilization failure, factors predictive of poor mobilization, and potential mobilization regimens for such patients.

## Introduction

Multiple myeloma (MM) accounts for 1% of all cancers and 10% of all hematologic malignancies [1]. High-dose therapy (HDT) followed by autologous hematopoietic stem cell transplantation (auto-HCT) is an important and potentially curative treatment modality for eligible patients with MM [2]. Besides, auto-HCT has been shown to increase the depth of response, progression-free survival (PFS), and overall survival (OS) in eligible MM patients [3]. Over the past decade, mobilized peripheral blood stem cells (PBSCs) have largely replaced bone marrow as the predominant source of repopulating hemopoietic stem cells (HSCs) for auto-HCT as they contain much larger numbers of CD34^+^ cells and offer convenient collection procedure and rapid hematologic recovery [4]. Moreover, to ensure successful multi-lineage engraftment after transplantation and sustained hemopoietic recovery, a minimal dose of 2 × 10^6^ CD34^+^ cells/kg body weight and an optimal dose of > 5 × 10^6^ CD34^+^ cells/kg are required for better post-transplantation clinical outcomes and sustained recovery [5]. However, the collection of sufficient autologous PBSCs relies on the successful mobilization of HSCs from the bone marrow niche into circulation. Therefore, successful HSCs mobilization is a crucial part of effective auto-HCT in patients with hematological malignancies including MM.

Common stem cell mobilization strategies include cytokine mobilization involving granulocyte colony-stimulating factor (G-CSF) or granulocyte–macrophage colony-stimulating factor (GM-CSF) alone; chemomobilization using chemotherapy/chemotherapy followed by cytokine administration (G-CSF); or G-CSF in combination with plerixafor, a selective CXCR4 cytokine receptor antagonist. These strategies differ in stem cell yields, safety considerations, resource utilization, and levels of contamination of the apheresis product with tumor cells [6]. In addition, new advances in effective mobilization of PBSCs have permitted a greater proportion of patients to benefit from auto-HCT. Various mobilization regimens seem to affect the graft cellular composition in patients with MM. For an instance, a higher number of lymphocytes content in the graft correlated with faster lymphocyte recovery after auto-HCT [7]. However, limited data are available on the effects of graft characteristics in patient outcomes other than hematopoietic engraftment. The current review comprehensively summarizes the associations between the content of PBSCs grafts and clinical outcomes, current options for HSCs mobilization, and potential strategies for managing initial poor mobilization/mobilization failures.

## Graft characteristics and effect on patient outcomes

Graft characteristics are important for auto-HCT recipients to ensure adequate hematopoietic engraftment and immune reconstitution [8]. Graft characteristics including CD34^+^ content, lymphocyte subsets, natural killer (NK) cells, and dendritic cells (DCs) will impact engraftment, immune recovery, and patient outcomes [8]. Further, several studies substantiated that graft characteristics may be important predictors for PFS and OS in patients receiving auto-HCT. Besides, existing mobilization strategies reported differences in graft characteristics and content. Therefore, it is pivotal to consider graft characteristics in autologous stem cell transplantation (ASCT) candidates with MM.

### CD34^+^ cell dose — role in engraftment and outcomes

The International Myeloma Working Group (IMWG) recommends that an average of 8 × 10^6^ CD34^+^ cells/kg should be given if mobilized, and that the minimum administration target should be 4 × 10^6^ CD34^+^ cells/kg progenitor cells for auto-HCT eligible MM patients [9]. The number of CD34^+^ cells has been considered the most important graft parameter. Recently, Elifcan et al. evaluated the relationship between the CD34^+^ hematopoietic progenitor cells dose and survival in MM patients who underwent auto-HCT and reported that the increase in the amount of CD34^+^ cells dose during HDT in MM patients shortened the platelet and neutrophil engraftment time and improved OS [10]. In a retrospective study with 508 MM patients, a threshold of 2.00–2.50 × 10^6^ CD34^+^ cells/kg in PBSCs transplantation was associated with adequate engraftment, but accelerated hematological reconstitution and reduced hospitalization with higher cell doses of ≥ 6.55 × 10^6^ cells/kg with selected CD34^+^ cells and ≥ 7.50 × 10^6^ cells/kg with non-selected CD34^+^ cells [11]. Similarly, Toor et al. reported the survival outcomes in MM patients (*N* = 104) undergoing a single transplant after conditioning with a conventional myeloablative regimen, busulphan, and cyclophosphamide and reported that higher CD34^+^ cell dose (> 4 × 10^6^ cells/kg) infused were independently predictive of improved OS and PFS [12].

Wahlin et al. evaluated the prognostic influence of pretransplant characteristics on response and survival in MM patients (*N* = 104) receiving uniform pretransplant treatment consisting of VAD (vincristine, doxorubicin, and dexamethasone) regimen, stem cell mobilization, and conditioning with melphalan 200 mg/m^2^ and reported that patients with higher harvest yields of CD34^+^ cells (> 11.8 × 10^6^ cells/kg) had better OS [13]. However, a higher yield of CD34^+^ cells (≥ 8 × 10^6^ CD34^+^ cells/kg) exhibited inferior PFS than those with low CD34^+^ cells collection in a large cohort of 621 MM patients and suggested that high stem cell collection does not correlate with better survival [14]. Hence, a conclusion cannot be drawn whether the higher CD34^+^ cell dose or CD34^+^ cells collection is associated with superior clinical outcomes and the rationale for this observation still remains elusive. Further substantiation in randomized clinical studies are warranted as all the previous evidences were from retrospective studies.

Though CD34^+^ cell is widely recognized as a biomarker reflecting PBSCs, the heterogeneity of subtypes makes it difficult to be considered as a desired indicator for long-term platelet engraftment. CD34^+^ CD33^−^ cell dose (> 1.38 × 10^6^ CD34^+^ cells/kg) was shown to better predict platelet recovery than CD34^+^ cell dose [15]. Another study reported higher CD34^+^ CD33^−^ cell doses to be correlated with rapid neutrophil recovery [16]. In addition, the primitive CD34^+^ CD38^−^ stem cells have been observed to affect engraftment following HDT as reported by Henon et al. where CD34^+^ CD38^−^ cell dose at 5 × 10^4^ cells/kg showed better and sustained engraftment when compared to low cell doses [17]. In contrary, another study showed no significant association of both CD34^+^ CD38^−^ and CD34^+^ HLA-DR^−^ cell dose with platelet count and long-term hematopoietic reconstitution [18]. Hence, the use of the number of more primitive stem cells as a marker of graft quality requires further validation.

### Lymphocyte content of the graft

A high-dose conditioning therapy before PBSC administration alters the immune system with a major impact on T-lymphocyte biology [19]. Studies have shown that in addition to the threshold number of CD34^+^ cells considered for an adequate PBSC collection, a certain number of lymphocytes should also be aimed for better outcomes [20]. Absolute lymphocyte count (ALC) reflects the restoration of hematological parameters after autologous PBSCs transplantation and is an independent prognostic factor for clinical outcomes in several hematological malignancies. This was evidenced in a phase III study by Porrata et al. as a higher autograft ALC (≥ 0.5 × 10^9^ lymphocytes/kg) was associated with better survival after ASCT in patients with non-Hodgkin lymphoma (NHL) [21]. The importance of collecting not only enough stem cells for hematologic engraftment but also enough immune effector cells (i.e., autograft ALC) to improve clinical outcomes in lymphoma patients post auto-HCT was highlighted in a case–control study [22]. In addition, a high autograft ALC of ≥ 0.5 × 10^9^ cells/kg showed improved clinical outcomes post-ASCT in patients with double/triple hit lymphomas [23]. Similarly, a retrospective study conducted by Hilmi et al. in newly diagnosed MM patients (*N* = 537) indicated that the MM patients with an ALC ≥ 1.4 × 10^9^/L experienced superior OS compared with an ALC < 1.4 × 10^9^/L (65 vs. 26 months, *P* < 0.0001) [24]. It is hypothesized that the dose of infused peripheral blood autograft lymphocytes is associated with early recovery of ALC post-ASCT which in turn is associated with improved outcomes. This relationship was established in a study by Porrata et al. where the ALC was found to be both a strong predictor for area under curve (AUC = 0.93;* P* = 0.0001) and strongly correlated with ALC at day 15 (ALC-15) recovery (*r*_s_ = 0.83; *P* = 0.0001). Furthermore, median post-transplant OS and time to progression (TTP) were longer in MM patients who received an ALC ≥ 0.5 × 10^9^ lymphocytes/kg when compared to those receiving ALC < 0.5 × 10^9^ lymphocytes/kg [25].

A retrospective analysis of ALC at different time points in patients with MM (*N* = 729) reported that ALC ≥ 1400 cells/μL or < 1400 cells/μL at post-auto-HCT at D0, D15, and D90 experienced a different OS (111, 90.7, and 84 months vs. 74, 70.5, and 65 months, respectively) [26]. Besides, Narwani et al. reported that after induction therapy at day 29, MM patients with an ALC > 0.8 × 10^9^/L had better OS compared with patients with an ALC-29 < 0.8 × 10^9^/L (58.3 vs. 42.5 months). The article further concluded that ALC at day 29 of treatment is a powerful predictor of outcome in MM [27]. In a nutshell, the infused dosage of autograft lymphocytes significantly impacts clinical outcome post auto-HCT in MM, via early recovery of post-ASCT ALC. However, there exists a heterogeneity regarding the predictive optimal threshold and timing of lymphocyte recovery as noted earlier. Hence, further studies evaluating the impact of ALC recovery on post HCT outcomes with examination of optimal ALC threshold and timeline are warranted.

Different subsets of autograft lymphocytes have been shown to be associated with post-ASCT prognosis in MM patients. For instance, the number of CD4^+^ and CD8^+^ T cells plays a role in predicting the prognostic outcomes of MM patients. In a study by Atta et al. a high CD8^+^ lymphocyte dose in the autograft was an independent predictor for early ALC recovery after ASCT, suggesting a critical role of CD8^+^ lymphocyte dose in the autograft for early lymphocyte recovery [28]. Schmidmaier et al. studied the influence of reinfused lymphocyte subsets on event-free survival (EFS) and OS in MM patients (*N* = 41) and reported that increased number of CD4^+^ cells and increased ratio of CD4/CD8 are significantly correlated with prolonged EFS [29]. In another study, Kaddoura et al. observed that patients with higher CD3 content had better PFS and OS suggesting a possible role of absolute CD3 and CD3/CD34 ratios in predicting clinical outcomes following ASCT [30]. Similarly, the infused dose of B cells can also predict the prognosis of MM patients. In a study by Lee et al., the cell doses of infused CD8^+^ (*P* = 0.042) T cells as well as CD19^+^ B cells (*P* = 0.044) were significantly associated with the ALC at engraftment [31]. Evidence in B cells dose associated with clinical outcomes is limited and currently does not support routine monitoring in clinical practice.

Data regarding graft NK cells and their role in post-transplant recovery in MM patients are limited. Compared to patients with low NK cells (< 100/uL), high NK cell count at 1 month after auto-HCT showed significantly prolonged PFS, suggesting a link between faster blood NK cell count recovery with improved outcome [32]. In addition to ALC, a 13-year follow-up of a phase 3 study showed that the infusion of NK cells was a predictor for OS and PFS, as both the outcomes were higher in patients receiving autograft NK ≥ 0.09 × 10^9^ cells/kg than < 0.09 × 10^9^ cells/kg [33]. However, in another study, a low graft NK cell count (< 2.5 × 10^6^/kg) did not significantly impact PFS (25 vs. 30 months, *P* = 0.155) or OS [34]. A higher pretransplant and post-transplant levels of DC are also known to be associated with improved OS in patients undergoing ASCT for relapsed or refractory NHL [35]. However, these observations require confirmation, especially in MM patients, as they seem to have important implications for mobilization strategies.

### Tumor cell contamination of the graft

Mobilization of myeloma cells and contamination of leukapheresis products by myeloma cells have been reported by different mobilization regimens. Moreover, patients with graft contamination (> 4.5 × 10^5^ plasma cells/kg) had a high risk of early disease progression following HDT [36]. Recently, Kostopoulos et al. prospectively revealed significant correlations between contamination of the stem cell graft and the depth of response achieved post-ASCT in MM patients (*N* = 199) with the highly sensitive next-generation flow (NGF) cytometry approach, suggesting graft contamination as a promising prognostic biomarker with independent predictive value for deeper response including minimal residual disease (MRD) negativity [37]. Significant reduction of tumor cells in the harvests can be obtained with repeated cycles of induction treatment before mobilization or by positive selection of CD34^+^ progenitor cells from the apheresis products [36]. The induction regimens are also likely to influence the autograft MRD status in patients with MM. A study by Bal et al. revealed a higher stem cell autograft purity/MRD-negativity with KRD (carfilzomib with lenalidomide and dexamethasone) than VRD (bortezomib with lenalidomide and dexamethasone) (81.4% versus 57.1%) [38].

However, there exist contrasting evidences on the influence of contaminating tumor cells in grafts and suggested mixed results in MM patients [39, 40]. Besides, these contrasting pieces of evidence might be due to differences in sensitivity of available testing and/or purging methodologies. MRD assessment is the most sensitive approach to measure the depth of response in MM patients, and persistent MRD after treatment indicates relapse in the near future. Therefore, MRD status in stem cell autografts has key prognostic implications. MRD assessment has been introduced in the IMWG, which recommends MRD tests for all MM patients who have achieved complete response [41]. Paiva et al. conducted a prospective analysis of the prognostic importance of MRD detection and reported that after auto-HCT, MRD^+^ MM patients had inferior PFS and OS compared with MRD^−^ patients [42]. Nevertheless, the impact of autograft tumor cell contamination on long-term safety and clinical outcome is still controversial as noted earlier. Previous studies have suggested no significant influence of graft contamination on survival or relapse risk [43]. Notably, most of the clinical studies were performed before the use of novel treatments and hence, in vivo tumor debulking may be much higher today with a higher potential of contaminated autografts and reinfused tumor cells inducing relapse. Therefore, a conclusive decision cannot be made on the role of residual plasma cells and ex vivo purging. Although MRD assessment has emerged as an integral component of MM treatment response assessment, the sensitivity of the MRD detection platform affects the prognostic value of MRD. MRD negativity determined by NGS and NGF, which are highly sensitive methods, had a better prediction of prognosis than that determined by a less sensitive method such as MFC.

### Effect of mobilization regimen on graft characteristics

Stem cell mobilization regimens that are used may have a different impact on graft characteristics which in turn have important long-term consequences for the patient. The effects of major mobilization regimens on graft characteristics are presented in Table 1. Most of the studies showed a significantly higher dose of lymphocytes with G-CSF alone than G-CSF plus cyclophosphamide [7, 44]. Furthermore, studies have proven the combination of G-CSF and plerixafor to be better than G-CSF alone with a significant increase in primitive CD34^+^ CD38^−^ cells by G-CSF plus plerixafor [45]. Mobilization regimen also seems to affect the tumor cell contamination which in turn influences survival [36, 39]. However, the evidence on the effect of the mobilization regimen on various lymphocyte subsets in the graft and tumor cell contamination is limited and requires further substantiation.

**Table 1:** Effect of mobilization regimen on graft characteristics

Graft characteristics	Mobilization regimen	Key observations	Reference
Lymphocyte content	Filgrastim, pegfilgrastim, and cyclophosphamide + filgrastim	-Mobilization with cyclophophamide reduces the number of mobilized and collected lymphocytes and NK cells as compared to mobilization with growth factors only -No difference in mobilization was observed between filgrastim and pegfilgrastim	[97]
	Hematopoietic growth factor (HGF) vs. HGF + cytoxan chemotherapy (C + HGF)	Mobilization with HGF had a higher absolute lymphocyte count compared to those mobilized with C + HGF [0.764 × 10^9^ lymphocytes/kg (range: 0.146–1.803) vs. 0.212 (range: 0.016–1.26), *P* < 0.0001]	[98]
	G-CSF cyclophosphamide 1–2 g/m^2^ plus G-CSF (LD-CY) Cyclophosphamide 3–4 g/m^2^ and G-CSF (ID-CY)	Significantly higher lymphocyte dose was obtained with G-CSF alone compared with the LD-CY and ID-CY groups	[99]
	G-CSF only vs. G-CSF and cyclophosphamide	-G-CSF only mobilization showed significantly higher lymphocyte count at day 15 post-infusion (*P* < 0.001) -G-CSF only was associated with significantly improved OS (aHR = 0.60, 95% CI: 0.39–0.92, *P* = 0.018)	[44]
	G-CSF plus plerixafor vs. cyclophosphamide plus G-CSF	The numbers of CD19^+^ B lymphocytes and NK cells were higher in G-CSF plus plerixafor group than cyclophosphamide plus G-CSF	[100]
	Cyclophosphamide plus G-CSF vs. G-CSF	There was a greater proportion of CD34^+^ CD38^−^ cells and higher numbers of T and B lymphocytes as well as NK cells in G-CSF alone arm	[7]
	G-CSF plus plerixafor vs. G-CSF alone	A significant increase in primitive CD34^+^ CD38^−^ cells was observed with G-CSF plus plerixafor when compared with G-CSF alone	[45]
Tumor cell contamination	CY + G-CSF + prednisone	Greater than 2.7- to 4.5-log reduction in contaminating MM cells was achieved	[101]
	G-CSF plus plerixafor	No evidence of tumor contamination in the apheresis product	[75]
	G-CSF plus plerixafor vs. G-CSF	Tumor cells are not enhanced in the peripheral blood or apheresis products of patients treated with GCSF plus plerixafor when compared with G-CSF alone	[102]
	Chemotherapy and filgrastim	High contamination group (> 4.5 × 10^5^ plasma cells/kg) had a significantly reduced OS (*P* = 0.012) compared to low contamination group	[39]
	Chemotherapy and filgrastim	Patients with graft contamination (> 4.5 × 10^5^ plasma cells/kg) had a high risk of early disease progression	[36]

*CY*, cyclophosphamide; *G-CSF*, granulocyte colony-stimulating factor; *NK*, natural killer cells

## Current options for hematopoietic stem cell mobilization

Several mobilization strategies including G-CSF which gives a predictable peak CD34^+^ level within 4–5 days and CT (usually a cyclophosphamide-containing regimen in combination with G-CSF) have been used and have their own benefits and limitations [46]. Mobilization with a novel reversible CXCR4 chemokine-receptor antagonist plerixafor is another effective strategy being used to mobilize HSCs. Plerixafor is indicated in combination with G-CSF to enhance mobilization of HSCs to the peripheral blood and has demonstrated efficacy in patients with MM and NHL [47]. CD34^+^ cells yield, mobilization failure rate, safety, and healthcare resource utilization vary across different regimens. A summary table on different mobilization regimens and failure rates with hematopoietic stem cell mobilization in MM is presented in Table 2.

**Table 2:** Mobilization regimens and failure rates with HSC mobilization in multiple myeloma

Author	Study type	Study population (N)	Regimen	Median CD34^+^cell yield (× 10^6^/kg)	Failure rates (% failure to mobilize at least 2 × 10^6^ CD34^+^ cells/kg)	Engraftment outcomes	
						NE Median days	PE Median days
Growth factor mobilization regimen							
Pusic et al. [74]	Retrospective	384	G-CSF 10 µg/kg/day	4.6	24	-	-
Chitra et al. [103]	Phase II	19	PEG 12 mg as a single dose	8.4	0	-	-
Danylesko et al. [104]	Phase II open-label	24	Lipegfilgrastim 6 mg	8.26	-	11	13
Chemomobilization (combined with G-CSF) regimen							
Andrew et al. [105]	Retrospective	398	G-CSF (preemptive plerixafor)	4.4	6.3	11 (10–22)	17 (10–60)
			CY + G-CSF (preemptive plerixafor)	13.6	5	11 (7–28)	15 (8–56)
Hamadani et al. [106]	Prospective	55	LD-CY + G-CSF	7.8	6	14	18
		68	ID-CY + G-CSF	24.9	0	10	17
Lin et al. [107]	Prospective	78	LD-CY	6.93	19.2	11	14
		5	CY/DOX	7.4	20	11	14.5
		10	G-CSF	3.61	50	12	12
Arora M et al. [108]	RCT	35	CY + G-CSF	16.6	12	13	-
		37	CY + GM-CSF	12.8	14.7	16	-
Jelinek et al. [56]	Retrospective	40	AraC + G-CSF	28.6	-	12	11
Zhu et al. [57]	Retrospective	128	VP-16 + AraC + G-CSF	28.32	-	11	11
Plerixafor-combined mobilization regimen							
*Preemptive plerixafor*							
Shah et al. [62]	Retrospective	344	G-CSF + P	8.29	2.7	11–12	21–23
Holig et al. [109]	Phase 2	37	G-CSF + P	3.7	-	18	17
Cid et al. [110]	Case series	30	G-CSF + P	4.2	-	18	19
Milone et al. [111]	Prospective	102	CY or DHAP + G-CSF + P	3.9	5	10	-
*Upfront plerixafor*							
JF DiPersio et al. [112]	RCT (phase 3)	148	P + G-CSF	12.97	-	11	18
		154	Placebo + G-CSF	7.31	-	11	18
Mark et al. [113]	Retrospective	78	G-CSF + P	9.8 (Mean)	-	-	-
A Schmid et al. [114]	Phase 2	10	VNB + P + G-CSF	10.6	0	11	14
		10	VNB + P	9.5	20	11	14
		10	P + G-CSF	9.4	0	11	15
		10	VBN + G-CSF	8.9	20	11	12
Ogunniyi et al. [87]	Retrospective	138	P + G-CSF	5.8	1.4	-	-

-: Not reported; *NE*, neutrophil engraftment; *PE*, platelet engraftment; *G-CSF*, granulocyte colony-stimulating factor; *CY*, cyclophosphamide; *GM-CSF*, granulocyte–macrophage colony-stimulating factor; *HSC*, hematopoietic stem cell; *VNB*, vinorelbine; *Eto*, etoposide; *LD-CY*, cyclophophamide 1–2 g/m^2^; *ID-CY*, cyclophophamide 7 g/m^2^; *IEV*, ifosfamide, etoposide, epirubicin; *CY/DOX-CY* + , doxorubicin, dexamethasone, G-CSF; *P*, plerixafor; *SC*, subcutaneous; *IV*, intravenous; *DHAP*, dexamethasone, cytarabine, cisplatin

### Cytokine alone

G-CSF has well-established kinetics and demonstrated favorable toxicity and cost profiles in MM patients undergoing auto-HCT. Further, there exists a discordance in G-CSF dose and CD34^+^ cell yield [48]. However, a G-CSF dose of 10 μg/kg/day is widely recommended and the most commonly used dose in clinical practice. Other growth factors such as GM-CSF, pegylated G-CSF, and Tbo-G-CSF have also been studied for PBSC mobilization in MM patients [49]. Pegfilgrastim is a pegylated form of G-CSF and is less commonly used than non-pegylated G-CSF. A randomized trial involving multi-dose regimen of pegfilgrastim evidenced a higher CD34^+^ cells yield on the first apheresis compared to G-CSF [50]. However, clinical experience showed predictable mobilization and similar yields with both pegfilgrastim and G-CSF [51]. Moreover, cost-effectiveness of pegfilgrastim in comparison to non-pegylated G-CSF needs to be determined. Growth factor mobilization regimens and failure rates in MM is summarized in Table 2. Cytokine mobilizations are associated with some limitations including its efficacy only in patients at low mobilization failure risk and when given alone, up to 35% of patients are unable to mobilize sufficient numbers of CD34^+^ cells/kg to ensure successful engraftment [5].

### Chemomobilization

Another option for PBSC mobilization is chemomobilization especially in patients with active disease as it offers both mobilizing effect and possible anti-tumor activity. Several studies illustrate the augmented efficiency of mobilizing regimens with additional reduction of graft contamination when containing both chemotherapy and hematopoietic growth factors [52, 53]. However, studies have demonstrated no impact on transplantation outcomes (complete response [CR] rate, time to progression [EFS or OS]) [54]. Contrastingly, the increase in peripheral blood hematopoietic progenitor cell yields is often accompanied by greater toxicity [55].

Myeloma-specific chemotherapy regimens that have been used for mobilization include CAD (cyclophosphamide, doxorubicin, dexamethasone) and PACE (platinum, doxorubicin, cyclophosphamide, etoposide), which are seldom used in clinical practice. Cyclophosphamide (CY) at a dose of 2–4 g/m^2^ in combination with G-CSF is commonly used and has been a successful mobilization technique [54].

The efficacy and safety of other chemomobilization regimes including cytarabine (AraC), etoposide (VP-16), and AraC + VP-16 + G-CSF combination have also been reported [56]. Moreover, AraC + G-CSF was also evaluated to be more efficient than CY + G-CSF as a stem cell mobilization regimen in MM patients [56]. Besides, a recent retrospective study evaluated the efficacy and safety of triplet regimen of VP-16 with AraC plus G-CSF as a novel mobilization regimen in MM patients and reported that this combination was highly efficient in high-risk MM patients who were referred for tandem ASCT [57]. Hematologic toxicity is the most common complication reported with chemomobilization and infection has been observed as the most common non-hematologic toxicity [56, 57]. Larger trials evaluating the comparative efficacy and safety of various chemomobilization regimens are much warranted.

### CXCR4 inhibitor — plerixafor (upfront; just in time or preemptive; remobilization)

Plerixafor is a selective and reversible CXCR4 inhibitor that acts by inhibiting the binding of SDF-1α to CXCR4 on hematopoietic stem cells which shows a synergistic effect on PBSC mobilization when administered in combination with G-CSF [58]. Several studies have shown lower mobilization failure rates, better achievement of collection targets, and fewer apheresis sessions with plerixafor plus G-CSF compared with G-CSF alone (Table 2). Based on these evidences, it is reasonable to consider upfront use of plerixafor in addition to G-CSF for hematopoietic stem cell mobilization in MM patients, especially those at high risk for mobilization failure.

Besides, plerixafor can be reserved for preemptive mobilization or for salvage after mobilization failure. The addition of plerixafor in an immediate rescue model showed efficient and safe after both G-CSF alone and chemomobilization with extremely high success rates [59]. The risk adaptive strategy of plerixafor use is based on the pre-apheresis peripheral blood CD34^+^ count or the CD34^+^ cell yield after the day’s collection. However, there is significant variability in PB CD34^+^ thresholds used, and the ideal threshold remains unclear. Moreover, there exists a disparity among expert guidelines on preemptive plerixafor in selected patients. The European Society for Blood and Marrow Transplantation recommends preemptive plerixafor in selected patients at a CD34^+^ count of 10/µL and the decision on use of plerixafor is based on the patient’s clinical history and clinician’s judgment when the CD34^+^ cell count is 10–20/μL [60]. Likewise, The United Kingdom consensus statement recommends plerixafor administration at a CD34^+^ count < 15/µL and consideration of administration at a CD34^+^ count between 15/µL and 20/µL depending on clinical circumstances [61]. The American Society for Transplantation and Cellular Therapy (ASTCT) guidelines recommend use of plerixafor with G-CSF in all patients scheduled for ASCT especially in patients at high risk of failure [46]. Therefore, consensus on algorithms to predict mobilization failure in order to identify which patients would best benefit from addition of plerixafor to the mobilization regimen is much warranted. In clinical practice, higher CD34^+^ thresholds for plerixafor administration are being investigated. A single center study analyzed plerixafor use at different CD34^+^ thresholds (< 15/µL, < 20/µL, and < 40/µL) and showed that 91% of patients received plerixafor at a threshold of 40/µL with significantly greater single day collection yields [62].

Though effective, plerixafor is associated with a high cost per single-use vial [63]. However, the cost-effectiveness of plerixafor has been demonstrated by multiple studies. In a single center study, Griel et al. observed that a single fixed dose of plerixafor in 67% of patients was cost-effective with successful CD34^+^ cells collection in preemptive and rescue settings [64]. Furthermore, a subsequent analysis confirmed that a single, fixed-dose plerixafor schedule may be sufficient, as significantly more patients underwent successful ASCT after receiving plerixafor (59.6% before plerixafor versus 90% after plerixafor, *P* < 0.001) [65]. The cost-effectiveness of upfront plerixafor with G-CSF was demonstrated by few studies. Retrospective analyses showed that upfront plerixafor with G-CSF has similar or reduced costs compared with cyclophosphamide plus G-CSF, in addition to the lower failure rates (6 to 12.5% versus 21 to 29%) defined differently by the studies [66, 67]. Interestingly, another study demonstrated upfront plerixafor to have higher cost than preemptive use of plerixafor ($28,448 vs. $24,852, respectively, *P* = 0.0315) [68]. Compared to on-demand use of plerixafor in selective patients, the routine upfront use of plerixafor in all patients is likely to be less cost-effective, hence, it is suggestible to use upfront plerixafor in patients requiring fewer collection days and higher collection yields [69]. The ASTCT guidelines also recommend the upfront use of plerixafor especially in patients with an unusually high CD34^+^ cell dose need, mostly to support two or more cycles of high-dose chemotherapy [70].

Various strategies can be applied to circumvent higher costs associated with plerixafor by administering the drug only once in a single fixed-dose, and reducing other expenses such as avoiding the requirement for additional apheresis sessions and reducing the need for a second myelosuppressive mobilization therapy [64]. Moreover, rather than the upfront plerixafor, the use of preemptive plerixafor where it is only administered to patients likely to fail mobilization is suggestible [68]. Even in preemptive setting, it is valuable to define intervention timing and the timing to stop the therapy. The United Kingdom consensus statement recommends that if patients’ CD34^+^ count does not increase to > 10 cells/µL after the initial dose, then the further use of plerixafor is not suggestible [61]. Furthermore, plerixafor use in patients with a peripheral CD34^+^ count of < 5 cells/µL is susceptible to a higher risk of plerixafor failure [71]. However, studies have demonstrated the efficacy of plerixafor in high-risk patients as well. Sanchez et al. retrospectively analyzed the effectiveness of plerixafor in patients with CD34^+^ count of < 3.5 cells/μL and showed that 63% of patients in this patient population reached the standard minimal collection target of 2.0 × 10^6^ CD34^+^ cells/kg [72]. Similar observations were noted in another study where a substantial proportion (62.7%) of patients with very low CD34^+^ counts (< 5 cells/μL) benefitted from the addition of plerixafor [73]. The evidence on plerixafor use in high-risk patients is controversial, hence, the risk and benefit should be carefully balanced before plerixafor administration in high-risk patients.

## Poor mobilization

The traditional mobilization strategies have failure rates of as high as 5–10% among patients with MM [6, 74]. Poor mobilization is commonly defined as a failure to achieve the target CD34^+^ yield of at least 2 × 10^6^ CD34^+^ cells/kg body weight [75]. GITMO (Gruppo Italiano Trapianto di Midollo Osseo–Italian Group for Stem Cell Transplantation) proposed a hierarchic model for the description of poor mobilization. According to this model, “proven poor mobilizers” is defined as mobilization failure (CD34^+^ cell peak < 20/µL peripheral blood) after adequate preparation (after 6 days of G-CSF 10 µg/kg alone or after 20 days of G-CSF > 5 µg/kg following chemotherapy) or a CD34^+^ cell yield of < 2.0 × 10^6^/kg body weight after three consecutive apheresis [76].

### Risk factors for poor mobilization

Several patients and treatment-related risk factors have been associated with reduced PBSC mobilization. Patient-related factors including older age, disease-related factors such as extensive BM involvement with malignancy, and treatment-related factors including prior radiotherapy involving marrow-rich sites, multiple lines of chemotherapy, history of prior alkylating agent, fludarabine, platinum-containing regimens, and history of mobilization failure have been linked to an increased risk of mobilization failure [77]. In addition, existing evidence substantiates the total number of prior chemotherapy cycles and previous treatment with melphalan is more influential in predicting poor mobilization than age, sex, or body weight among MM patients [78]. Besides, prolonged initial therapy with a lenalidomide-based regimen, particularly in patients receiving G-CSF alone for mobilization, is known to impair hematopoietic stem cell collection [79], due to localization of CXCR4 to the cell surface and eventually blocking the mobilization of CD34^+^ cells induced by lenalidomide [80]. Recently, concerns have been raised against daratumumab that it could also be associated with poor mobilization in patients with newly diagnosed MM patients eligible for ASCT [81]. In addition, daratumumab-based induction before ASCT was also found to be associated with slightly increased risk of infectious complications, antibiotics use, and a slightly delayed hematopoietic recovery and also required more transfusions in patients with MM [82]. According to current available data, all these agents may have a variable impact on mobilization and collection.

### Strategies to deal with poor mobilizers in MM

Over the past decade, technical advancement in the stem cell mobilization strategies has offered the possibility to convert “poor mobilizers” into “good mobilizers.” Based on the current accumulated literature, several strategies including the use of plerixafor, larger volume leukapheresis (LVL), remobilization, and chemomobilization in combination with plerixafor and G-CSF are considered important strategies to increase the apheresis yields in poor mobilizers.

#### Upfront and preemptive plerixafor

The clinical efficacy and synergistic effect of the combination of plerixafor (upfront, preemptive) with G-CSF/chemo on PBSC mobilization has been discussed earlier. Recently, Cheng et al. conducted retrospective analysis in MM patients and reported that plerixafor was effective when given either preemptively or as a rescue strategy in poor mobilizers [83].

##### Proven poor mobilizers: preemptive plerixafor

Plerixafor is added in the preemptive approach for patients with poor mobilizers who were identified based on pre-apheresis CD34^+^ cell count. After administration of plerixafor, studies have reported almost fourfold increase in CD34^+^ cells and a high mobilization rates of > 90% [59]. Preemptive plerixafor use requires monitoring of PB CD34^+^ cell counts at the time of hematopoietic recovery for prediction of poor mobilization as well as identification of peak mobilization [84].

##### Predicted poor mobilizers: upfront plerixafor

Several approaches with the use of plerixafor have been investigated in predicting mobilization failure based on patients’ baseline characteristics [70]. A low failure rate (~ 4%) was observed when plerixafor was administered to predicted poor mobilizers based on baseline characteristics [70]. In addition, some risk-adapted algorithms for the earlier identification of PBSC mobilization failure and optimal utilization of plerixafor have been proposed. High-risk patients who had received 3 or more lines of prior chemotherapy, hype-CVAD, and 4 or more cycles of lenalidomide also benefited from upfront plerixafor and G-CSF combination, which showed significantly improved mobilization (larger proportion of patients collected 2 × 10^6^ CD34^+^ cells/kg in one apheresis) when compared to G-CSF alone [85]. Moreover, multiple studies have also shown the efficacy of upfront plerixafor in real-world settings as the addition of plerixafor to G-CSF mobilization provided improved CD34^+^ yield [86, 87].

##### Plerixafor combined with chemomobilization

Chemomobilization in combination with G-CSF is an alternative PBSC mobilization strategy for use in poor mobilizers undergoing auto-HCT. Chemotherapy-based mobilization strategies take a longer time (11–13 days) and require greater resource utilization. The addition of plerixafor to a chemotherapy + G-CSF mobilization regimen could increase PBSCs yield thereby decreasing the number of apheresis procedures required to collect an adequate number of PBSCs for transplantation [88]. Existing data support the use of preemptive plerixafor to salvage chemomobilization + G-CSF patients who failed to mobilize sufficient PB CD34^+^ cells, or who demonstrated declining PB CD34^+^ cell counts during apheresis. One small pilot study involving upfront chemomobilization plus plerixafor plus G-CSF in patients with MM and NHL demonstrated efficacy with a twofold increase in CD34^+^ cell collection [89]. Although most of the existing evidence on plerixafor in chemomobilization + G-CSF involves small single-center retrospective studies or case reports, majority of them reported successful collections. However, further evaluation in prospective trials is much needed.

#### Larger volume leukapheresis

Generally, stem cell apheresis is usually performed as a lower-volume procedure (volume of 10–15 L). LVL procedure with larger volumes (15–30 L) is also applied in selected circumstances. There exists contrasting evidence from studies investigating the performance of LVL with some studies showing that prolonged session of leukapheresis leads to an increased CD34^+^ cell yield per apheresis session, while others report the opposite [90]. In addition, the use of LVL in patients with a pre-apheresis peripheral blood CD34^+^ cell count of < 20 × 10^3^/mL may provide a 40–100% increase in PBSC yield. Zdenka et al. conducted a comparative study involving well-mobilized donors, well-mobilized patients, and weakly mobilized patients with hemato-oncological disease and reported that selected poor mobilizers may benefit from LVL [91]. Though the efficiency of LVL has been established, there still remains the question of safety as the larger volume of infused anticoagulants can cause hypocalcemia, metabolic alkalosis, hypokalemia, hypomagnesaemia, and a more pronounced thrombocytopenia. Generally, AEs associated with LVL are manageable. In one study involving 30 patients with hematological malignancies on whom LVL was performed, only mild symptoms of citrate toxicity were observed with only one patient experiencing mild perioral paresthesia of grade 1 [90]. Furthermore, LVL was safe even in small children, with only mild symptoms of citrate-induced hypocalcemia observed in two children. Even though there was a significant decrease in the platelet count after each procedure, no bleeding events were observed and there was no need for transfusion support [92].

#### Remobilization

Remobilization is a reasonable option for patients who have failed mobilization in the first attempt or patients with suboptimal CD34^+^ cell yield. A retrospective study involving patients who underwent stem cell mobilization for auto-HCT using predominantly G-CSF for remobilization (> 90% of cases) reported a failure rate of 81.6% for patients remobilized with G-CSF alone, whereas failure rates of 73.5% and 27.8% were reported in patients remobilized with chemotherapy plus G-CSF and G-CSF plus plerixafor, respectively [74]. Therefore, to overcome the high rate of remobilization failure, a higher dose of G-CSF up to 16–32 μg/kg/day has been evaluated and reported to increase the CD34^+^ cell yields [93, 94]. However, this high-dose strategy was associated with increased toxicity and cost. Therefore, it is evident that remobilization attempts using cytokine-only strategies do not effectively increase the PBSC yields in poor mobilizers. Although chemomobilization is an acceptable remobilization strategy for patients who have failed cytokine-only mobilization, high failure rates (74%) and greater toxicities associated with chemomobilization limit its application [74]. Among the currently available remobilization options, mobilization with plerixafor + G-CSF is associated with the lowest failure rates (< 30%) [95, 96]. Therefore, a remobilization regimen including plerixafor is recommended as an effective salvage option for patients who have experienced mobilization failure.

#### New mobilization agents

Several novel and experimental agents that may be useful in mobilizing PBSC are being analyzed and are at various phases of clinical development. A summary of new agents that enhance stem cell mobilization is presented in Table 3.

**Table 3:** New agents that enhance stem cell mobilization

S. no	Agent	Mechanism	Study phase	Key finding	Reference
1	Teriparatide	Stimulation of niche osteoblasts which in turn release endogenous G-CSF	Phase 1/2	Adequate mobilization in 47% of patients who failed 1 prior mobilization and 40% of patients who failed 2 prior mobilization attempts	[115]
2	Bortezomib	Alteration of the VLA-4/VCAM-1 pathway	Phase 3	Successful mobilization of 85% of patients treated with a bortezomib-based induction regimen	[116]
3	POL6326 (balixafortide)	Inhibition of CXCR4	Phase 2	Sufficient mobilization in 66% newly diagnosed myeloma patients	[117]
4	CDX-301	FLT3 agonist	Phase 1	DX-301 resulted in effective peripheral expansion of monocytes, hematopoietic stem and progenitor cells, and key subsets of myeloid DCs and plasmacytoid DCs, in healthy volunteers	[118]
5	TG-0054 (burixafor)	CXCL12/CXCR4 modulators	Phase 2	Burixafor in combination with G-CSF was able to mobilize > 5.0 × 10^6^ CD34^+^ cells/kg in 1–2 leukapheresis sessions	[119]
6	BKT 140 (BL8040)	CXCR4 antagonist	Phase 3	G-CSF + BL-8040 significantly increased the proportion of patients mobilizing ≥ 6 × 10^6^ CD34^+^ cells/kg for ASCT (92.5%) vs. G-CSF alone (26.2%)	[120]

Successful mobilization defined as patients with at least 2 × 10^6^ CD34^+^ cells/kg within defined apheresis days

*DCs*, dendritic cells; *G-CSF*, granulocyte colony-stimulating factor; *VLA-4*, very late activation antigen-4; *VCAM-1*, vascular cell adhesion molecule

## Conclusion

In summary, PBSC has largely replaced bone marrow as a source of stem cells for both autologous and allogeneic stem cell transplantation. Numerous studies have demonstrated associations between the content of PBSC grafts and clinical outcomes. However, extensive randomized clinical trial data substantiating the association between graft characteristics and clinical outcomes in MM is lacking. To manage poor mobilizers, the identification of risk factors is critical to decrease mobilization failure and avoid remobilization. Several studies are ongoing to identify new agents/combinations to enhance the efficacy of stem cell mobilization strategies, especially in those patients who are at risk for mobilization failure. Moreover, we believe that with the development of novel agents under trials, PBSC mobilization especially in poor mobilizers might be less challenging in the near future.

## Data Availability

The datasets generated during and/or analyzed during the current study are available from the corresponding author on reasonable request.
